# Prediction of KRAS gene mutations in colorectal cancer using a CT-based radiomic model

**DOI:** 10.3389/fmed.2025.1592497

**Published:** 2025-05-12

**Authors:** Wenjing Wang, Qingbiao Zhang, Shimei Fan, Yuyin Wang, Xingyan Le, Min Ai, Chunqi Du, Junbang Feng, Chuanming Li

**Affiliations:** ^1^Medical Imaging Department, Chongqing Emergency Medical Center, Chongqing University Central Hospital, School of Medicine, Chongqing University, Chongqing, China; ^2^Physical Examination Center, Chongqing Emergency Medical Center, Chongqing University Central Hospital, School of Medicine, Chongqing University, Chongqing, China; ^3^Department of Anesthesiology, Nanan District People's Hospital of Chongqing, Chongqing, China

**Keywords:** colorectal cancer, KRAS gene mutation, computed tomography, radiomics, machine learning

## Abstract

**Background:**

Determining the KRAS gene mutation status in colorectal cancer (CRC) before surgery is highly important for an individualized clinical treatment. This study aimed to explore the clinical value of radiomics models based on CT images in predicting the KRAS mutation status in patients with CRC.

**Methods:**

A total of 201 CRC patients who underwent surgery and pathology examinations from March 2022 to January 2025 were included. They were randomly allocated to a training group (160 patients) or a testing group (41 patients) at a ratio of 8:2. All patients underwent plain CT and contrast-enhanced examinations before surgery. The 3D segmentation of the tumour was manually delineated by two radiologists who were unaware of the pathological results and KRAS gene detection outcomes. The PyRadiomics package in Python was used to extract 2,264 radiomic features from each ROI. After dimensionality reduction, machine learning methods such as extremely randomized trees (ERT), random forest (RF), XGBoost, Bagging, and CatBoost were used for model construction. The performance of the models was compared using the area under the receiver operating characteristic curve (AUC), accuracy, sensitivity, and specificity. The Delong test was employed to assess the differences between the various models.

**Results:**

After feature selection, the top 8 features with the highest mutual information scores were extracted to construct a prediction model. The Delong test revealed that the XGBoost model, which is based on CT images from the vein phase, performed the best, with AUC values of 0.90 and 0.81 in the training and test sets, respectively. The calibration curve indicated a high consistency between the actual and predicted probabilities of the samples. The decision curve analysis results revealed that the XGBoost model exhibited the highest net clinical benefit among all the models.

**Conclusion:**

In this study, a highly accurate radiomics model was developed for KRAS gene mutation status prediction in patients with CRC before surgery. This technique avoids the potential risks of tumour rupture and dissemination during biopsy and can serve as a powerful tool to assist doctors in developing personalized and precise targeted treatments for colorectal cancer, which highly important in clinical work.

## Introduction

Colorectal cancer (CRC) is the third most common cancer globally, accounting for approximately 9.6% of new cancer cases and 9.3% of deaths annually, making it the second most common cause of cancer-related deaths (1). In recent years, the incidence and mortality rates of CRC have been increasing annually, imposing substantial health and economic burdens on patients and their families (2, 3). Therefore, exploring the pathogenesis of CRC and developing new therapeutic targets are clinically significant. Recently, the rapid development of molecular biology and genomics technologies has revealed the molecular characteristics of CRC, suggesting the importance of implementing an individualized treatment and precision medicine. For example, the Kirsten rat sarcoma (KRAS) gene is one of the most common mutated oncogenes in CRC, accounting for approximately 30%–50% of mutations (4, 5). KRAS mutations can cause a sustained activation of the RAS–RAF–MAPK signalling pathway, promoting the proliferation of tumour cells and leading to an ineffective treatment with anti-EGFR monoclonal antibodies (6). Anti-EGFR-targeted drugs have long been considered effective only for patients with wild-type KRAS, whereas KRAS-mutant drugs are considered ineffective (7, 8). Therefore, predicting the KRAS gene mutation status in CRC is highly important for individualized clinical treatment.

Currently, the preoperative prediction of KRAS mutations in CRC primarily relies on obtaining pathological samples via invasive needle biopsy (9, 10). However, this method is largely limited by the location and quantity of samples, making it difficult to comprehensively reflect the overall state of the tumour. In addition, needle biopsy may lead to tumour rupture, increasing the risk of tumour spread and metastasis. Liquid-based testing for KRAS status currently faces limitations such as an insufficient specificity and high testing costs (11). There are also studies on the use of MRI or PET to predict KRAS mutations in CRC; however, issues such as abdominal motion artefacts, high costs and ionizing radiation have greatly limited their clinical applications (12, 13). Computed tomography (CT) is the preferred imaging method for the clinical diagnosis of CRC owing to its significant advantages, such as rapid, wide-range scanning and multi-directional imaging. It can clearly display the thickening of the intestinal wall and assess the relationship between the tumour and surrounding blood vessels and organs, which is indispensable for the preoperative evaluation of CRC (14). Radiomics, first proposed by Lambin et al., can analyse the relationships among images, genes, and clinical information of disease classification, treatment efficacy, and prognosis prediction by deep mining high-throughput information of imaging data (15). It has demonstrated to be significant in disease screening, biopsy guidance, treatment-plan development, and prognosis evaluation (16–18), which can greatly help with the personalized and precise treatment of diseases. Therefore, this study aimed to explore the clinical value of radiomics based on CT images in predicting the KRAS mutation status in patients with CRS.

## Materials and methods

### Patients

This was a retrospective study that was approved by the ethics committee of our institution, and the requirement for written informed consent was waived.

**Figure 1:**
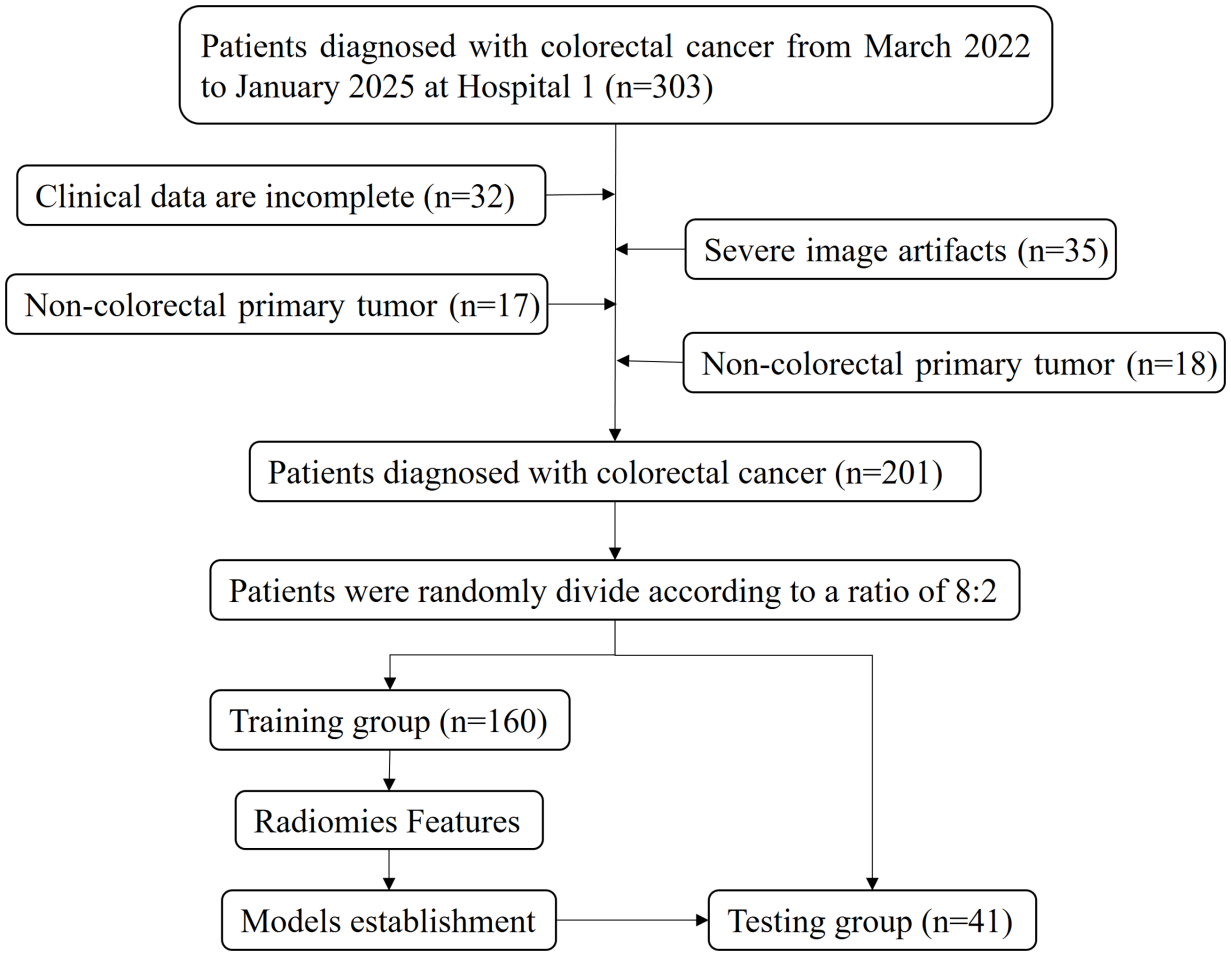
The flow chart of the study design.

A total of 201 CRC patients who underwent surgery and pathology examination at the Affiliated Central Hospital of Chongqing University from March 2022 to January 2025 were included. They were randomly allocated into training (160 patients) and testing (41 patients) groups at a ratio of 8:2 for model construction and validation. The inclusion criteria were as follows: (1) patients with CRC diagnosed with postoperative pathology and who underwent KRAS gene testing; (2) patients who underwent abdominal CT enhancement examination within 14 days before surgery; and (3) patients who did not receive radiotherapy, chemotherapy, or chemoradiotherapy before pathology examination. The exclusion criteria were as follows: (1) poor CT imaging quality; (2) incomplete clinical data; and (3) other concurrent malignancies. Clinical data such as age, gender, family history, hypertension status, diabetes status, carcinoembryonic antigen (CEA) levels, pathological results, and KRAS mutation results were collected from the electronic medical record system. The flow chart of the participant recruitment process is shown in Figure 1.

### CT imaging

All CT images were obtained using the New Revolution CT system by General Electric Co., Ltd., and the uCT760 system by United Imaging Co., Ltd. The patients were placed in a supine position with both arms raised, and the body centred on the bed surface. After inhalation, breath-holding scanning was performed, ranging from the diaphragmatic dome to the inferior margin of the pubic symphysis. Automatic tube current modulation technology was employed, with a rotation time of 0.5 s and a pitch of 0.992:1. The images were reconstructed using the adaptive statistical iterative algorithm (ASiR-V, weighted at 50%) and standard reconstruction convolution kernels, with a reconstruction thickness of 1.25 mm. Firstly, non-enhanced phase (NP) scans were performed, followed by three-phase contrast-enhanced scans after the injection of a contrast medium. The delay times were set as 25–30 s for the arterial phase (AP), 50–70 s for the venous phase (VP), and 120 s for the equilibrium phase (EP). The contrast medium was injected through the right median cubital vein with an 80-mL bolus of iodinated contrast medium (Iohexol, GE Pharmaceuticals Co., Ltd., 300 mgI/mL) at a rate of 3.0 mL/s, followed by a 30-mL bolus of saline at the same rate. The delay time was measured using the Smart Prep method, and the monitoring level was set at the hepatic hilum level. The trigger was placed at the descending aorta, with a trigger threshold of 180 Hu.

### Tumour segmentation

3D segmentation of the tumour was manually delineated by two radiologists with over 20 years of experience utilizing the Intelligent Medical Research Platform (Version 20240130, United Imaging Co., Ltd., Shanghai, China). The pathologists were unaware of the pathological results and KRAS gene detection outcomes. Previous studies on enhanced CT images of gastrointestinal tumours revealed that the internal structural details and boundaries of the tumour were best displayed in the VP images (19, 20). Therefore, in this study, we first outlined the region of interest (ROI) in the VP phase (window level: 40 window width: 300) for the entire tumour (including the abnormally thickened and enhanced intestinal wall) while avoiding the intestinal contents and gas. Subsequently, we copied the ROI from the VP to the NP phase.

### Radiomics feature extraction and feature selection

First, image preprocessing was performed, including grayscale discretization (binwidth = 25), image normalization (window width and level normalization), and image resampling (interpolation method selected as ‘BSpline’, resampling interval set to [1,1,1]) (21). Then, the PyRadiomics package in Python (version 2.1.2, https://pyradiomics.readthedocs.io/) (22) was used to extract radiomic features from the NP and VP images. Each ROI contained 2,264 radiomics features, including a first-order histogram, 3D morphology, grey level co-existence matrix (GLCM), grey level range-matrix (GLRM), grey level size zone matrix (GLSZM), neighbouring grey tone difference matrix (NGTDM), and grey level dependence matrix (GLDM) features. The standard scaler method was employed to standardize the data. This step transformed the original data into a standard normal distribution with a mean of 0 and a standard deviation of 1, thereby eliminating the differences in dimensions and scales among the different features and ensuring a reasonable weight distribution for each feature in the subsequent analysis. For the feature selection step, this study utilized the recursive feature elimination (RFE) method, which is based on a random forest kernel. This method leverages the evaluation capability of the random forest model for feature importance, gradually selecting a subset of features that have a key impact on the target variable by recursively removing the least important features, thereby effectively reducing data dimensionality and improving the model training efficiency and generalization ability. Additionally, the mutual information method, which can measure the degree of mutual dependence between two variables, was used to filter the features. By calculating the mutual information score between each feature and the target variable (KRAS mutation status), features with scores higher than 0.05 were selected. Among these selected features, those with higher mutual information scores were further chosen to capture key information related to the KRAS mutation status and to construct a model to achieve an accurate determination of the KRAS mutation status.

### Model construction and evaluation

**Figure 2:**
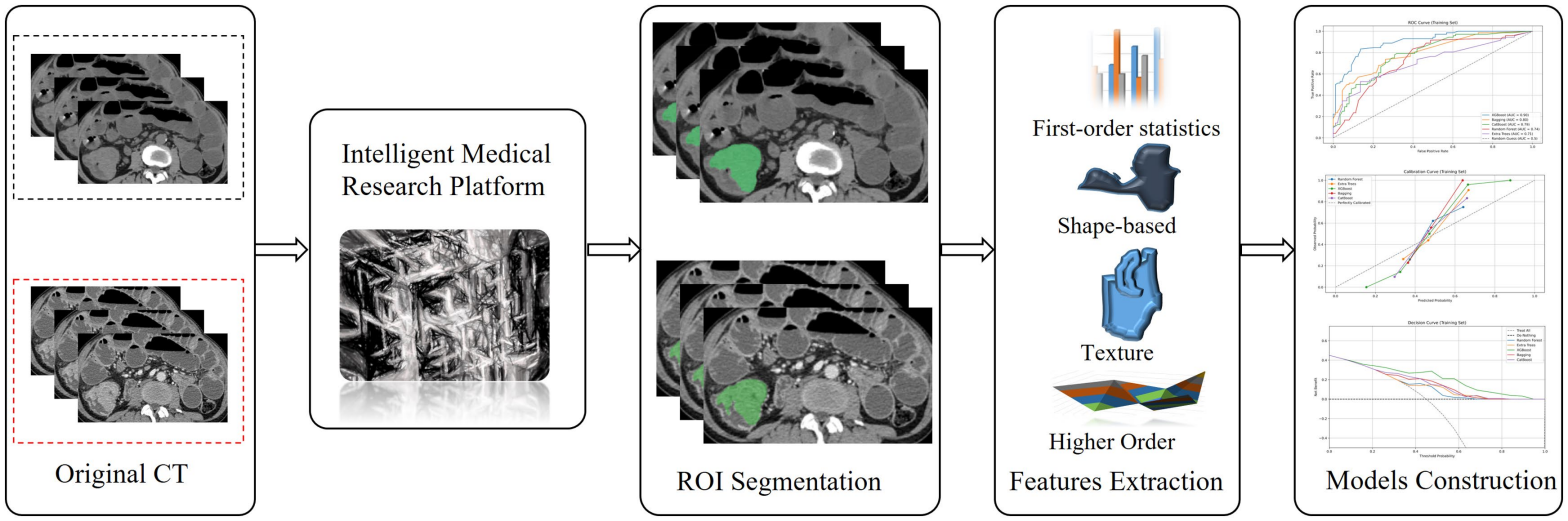
The flow chart of colorectal cancer segmentation, feature extraction and models Construction.

Machine learning methods, namely extremely randomized trees (ERT), random forest (RF), XGBoost (XB), bagging, and CatBoost, were used for model construction. The optimal parameters for each algorithm were selected using 10-fold cross-validation to establish the VP, NP, and VP-NP models. The performance of the models was compared using the area under the receiver operating characteristic curve (AUC), accuracy, sensitivity, and specificity. The Delong test was employed to assess the differences between the various models. The relationship between the predicted and actual probabilities was evaluated using a calibration curve, and the net benefit of the model for patients was predicted using a decision curve. The architecture of the core process of this study is clearly presented in Figure 2.

### Statistical analysis

The data analysis was conducted using the SPSS 25.0 statistical software. Quantitative data with a normal distribution are expressed as the mean ± standard deviation. Independent sample *t*-tests were used when the variables followed a normal distribution, and Mann–Whitney U tests were applied when they were non-normal. Chi-square tests were employed to analyse the qualitative variables to determine whether the differences were statistically significant. All the statistical tests were two-tailed, and a *p*-value of < 0.05 was considered statistically significant.

## Results

This study included a total of 201 patients, 103 males and 98 females, with an average age of 67.90 ± 11.26 years ranging from 24 to 94 years. According to the KRAS gene test results, the patients were divided into a KRAS mutation group (*n* = 92, 45.77%) and a KRAS wild-type group (*n* = 109, 54.23%). In the KRAS mutation group, 40 males (43.48%) and 52 females (56.52%) were included, whereas in the KRAS wild-type group, 63 males (57.80%) and 46 females (42.20%) were included. There was a statistically significant gender difference between the two groups (*p* = 0.043). Compared with that in patients in the KRAS wild-type group, the median CEA level was higher in those in the KRAS mutation group (5.61 [2.91, 15.95] vs. 3.24 [1.91, 6.45], *p* = 0.001). There were no statistically significant differences in age, family history, history of hypertension, history of diabetes, tumour location, or the tumour pathological stage between the two groups (*p* > 0.05) (Table 1).

**Table 1:** Patient characteristics in the training set and testing set.

Characteristics	Total group	Wild-type group	Mutated group	*p*-value
	(*n* = 201)	(*n* = 109)	(*n* = 92)	
Age, years (Mean ± SD)	67.90 ± 11.26	68.35 ± 11.09	67.36 ± 11.49	0.417
Gender, *n* (%)				0.043*
Male	103 (51.24)	63 (57.80)	40 (43.48)	
Female	98 (48.76)	46 (42.20)	52 (56.52)	
Family history, *n* (%)				0.911
No	184 (91.54)	100 (91.74)	84 (91.30)	
Yes	17 (8.46)	9 (8.26)	8 (8.70)	
CEA (Median [Q1,Q3])		3.24 [1.91,6.45]	5.61 [2.91,15.95]	0.001*
Hypertension, *n* (%)				0.055
No	112 (55.72)	54 (49.54)	58 (63.04)	
Yes	89 (44.28)	55 (50.46)	34 (36.96)	
Diabetes, *n* (%)				0.698
No	149 (74.13)	82 (75.23)	67 (72.83)	
Yes	52 (25.87)	27 (24.78)	25 (27.17)	
TNM stage, *n* (%)				0.311
I	11 (5.47)	5 (4.59)	6 (6.52)	
II	78 (38.80)	44 (40.37)	34 (36.96)	
III	84 (41.79)	41 (37.66)	43 (46.74)	
IV	28 (13.93)	19 (17.43)	9 (9.78)	
Location, *n* (%)				0.226
Right colon	48 (23.88)	22 (20.18)	26 (28.26)	
Left colon	75 (37.31)	46 (42.20)	29 (31.52)	
Rectum	78 (38.81)	41 (37.62)	37 (40.22)	

*p* value was calculated from two-sample *t* test for continuous variables and from Chi-squared test for discrete variables. *p values less than 0.05 were considered as statistically significant.

**Figure 3:**
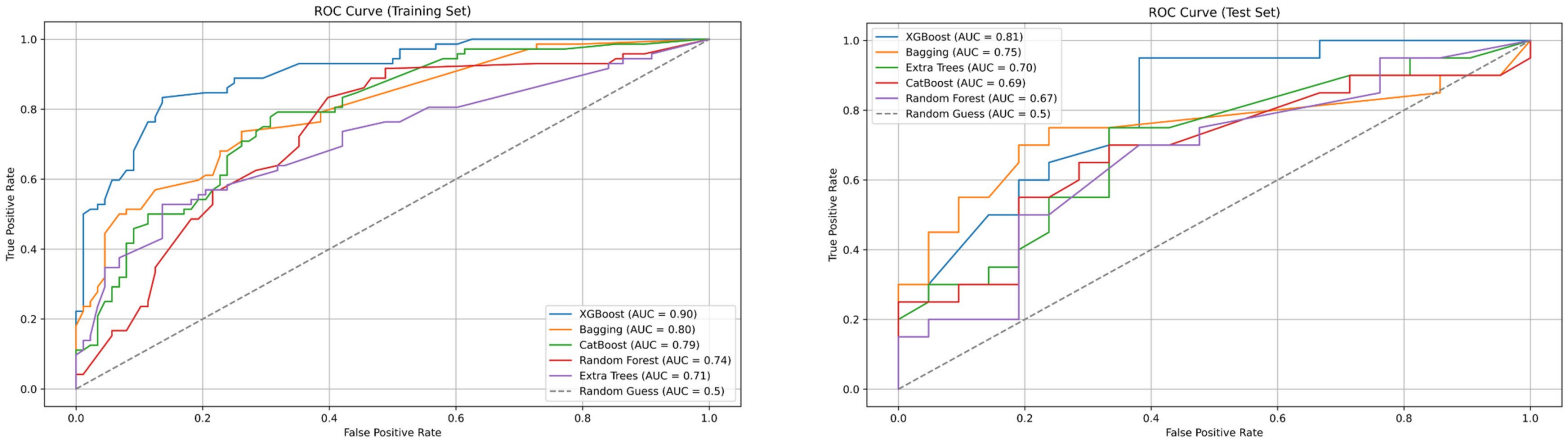
Receiver operating characteristic (ROC) curves of XGBoost, Bagging, CatBoost, Random Forest, Extra Trees and Random Guess models in the training and testing dataset base on venous phase CT.

**Figure 4:**
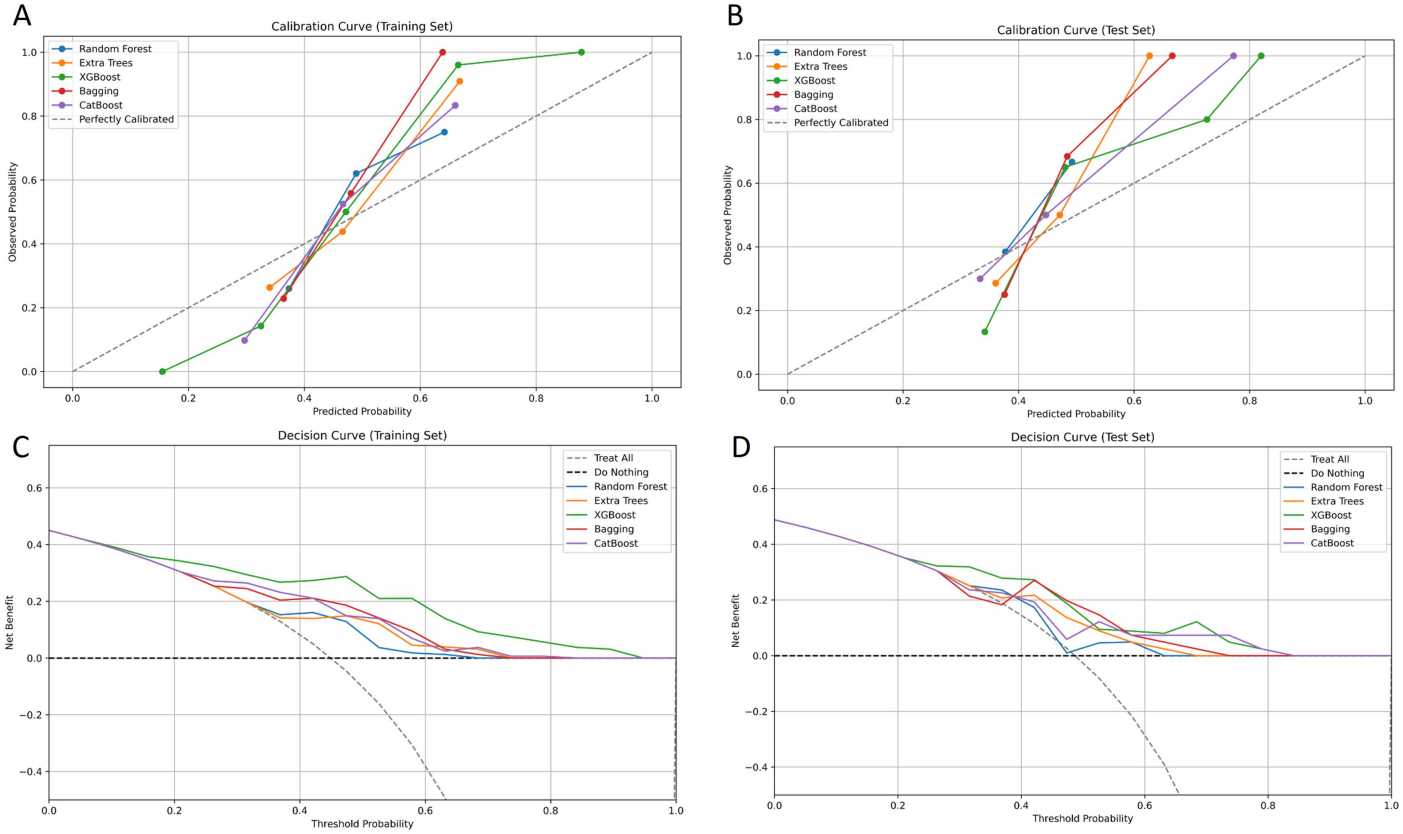
Calibration curves of goodness-of-fit for the training set and the test set. The 45° line in the figure serves as an ideal reference, representing the situation where the predicted probabilities of the model are completely consistent with the actual probabilities. The closer the calibration curve of the model is to the 45° line, the higher its prediction accuracy **(A, B)**. Decision curves of different models for the training set and the test set. The y-axis of the decision curve reflects the net benefit, and the x-axis represents the threshold probability. Through comparison, it is found that the XGBoost model demonstrates a higher overall net benefit than other models when predicting KRAS gene mutations in colorectal cancer patients **(C, D)**.

A total of 2,264 radiomic features were extracted from each ROI in the VP and NP images. The features were filtered using the recursive feature elimination (RFE) method, which is based on a random forest kernel, resulting in 225 and 226 features in the VP and NP images, respectively. The features with mutual information scores higher than 0.05 were then selected using the mutual information method, resulting in 26 and 28 features in the two phases, respectively. The top 8 features with the highest mutual information scores were extracted to construct a KRAS mutation prediction model (Table 2). The performances of RF, BG, ET, XB, and CB are shown in Table 3. The Delong test revealed that the XB model based on the VP phase images performed the best, with AUC values of 0.90 and 0.81 in the training and test sets, respectively (Figure 3). The sensitivity, specificity, and accuracy in the training and test sets were 0.848, 0.896, and 0.874, and 0.870, 0.852, and 0.860, respectively. The calibration curve indicated a high consistency between the actual and predicted probabilities of the samples (Figures 4A,B). The analysis of the decision curve results revealed that the XB model exhibited the highest net clinical benefit among all the models (Figures 4C, 4D).

**Table 2:** Features retained after dimensionality reduction by recursive feature elimination.

ROI	Features
Non-enhanced phase	mean_firstorder_RobustMeanAbsoluteDeviation;
	wavelet_ngtdm_wavelet-LHL-Busyness;
	log_glszm_log-sigma-2-0-mm-3D-LowGrayLevelZoneEmphasis;
	wavelet_firstorder_wavelet-LHL-Kurtosis;
	wavelet_glszm_wavelet-LLH-ZoneEntropy;
	mean_glcm_Correlation;
	wavelet_firstorder_wavelet-LLH-Kurtosis;
	normalize_gldm_DependenceVariance

Venous phase	specklenoise_firstorder_Minimum;
	binomialblurimage_glrlm_RunLengthNonUniformityNormalized;
	log_glcm_log-sigma-2-0-mm-3D-ClusterProminence;
	curvatureflow_firstorder_10Percentile;
	curvatureflow_firstorder_Kurtosis;
	binomialblurimage_glszm_HighGrayLevelZoneEmphasis;
	boxsigmaimage_firstorder_Kurtosis;
	binomialblurimage_firstorder_RobustMeanAbsoluteDeviation;

**Table 3:** Results of random forest (RF), bagging (BG), extra trees (ET), xgboost (XB) and catboost (CB) models on training set and testing set.

Models	Phase	Training Set|Testing Set		
		AUC	Sensitivity	Specificity
RF	VP	0.74 (0.65–0.81)|0.67 (0.48–0.82)	0.93|0.95	0.19|0.24
	NP	0.65 (0.57–0.73)|0.65 (0.46–0.80)	0.81|0.85	0.00|0.00
BG	VP	0.80 (0.73–0.86)|0.75 (0.54–0.88)	0.75|0.75	0.67|0.76
	NP	0.63 (0.54–0.72)|0.59 (0.40–0.75)	1.00|1.00	0.00|0.00
ET	VP	0.71 (0.63–0.79)|0.70 (0.51–0.84)	0.81|0.75	0.44|0.67
	NP	0.74 (0.65–0.81)|0.71 (0.52–0.86)	0.65|0.70	0.67|0.81
XB	VP	0.90 (0.85–0.94)|0.81 (0.64–0.92)	0.93|0.95	0.58|0.62
	NP	0.69 (0.61–0.77)|0.56 (0.37–0.74)	0.94|1.00	0.19|0.29
CB	VP	0.79 (0.72–0.86)|0.69 (0.49–0.84)	0.79|0.70	0.61|0.67
	NP	0.63 (0.55–0.71)|0.53 (0.35–0.69)	1.00|1.00	0.00|0.00

## Discussion

KRAS mutations are common in colorectal cancer (30%–50%) and are considered important molecular markers for predicting the efficacy of the anti-EGFR monoclonal antibodies, cetuximab, and panitumumab (23, 24). For CRC patients who have metastasis or are unable to undergo a complete resection, an accurate and noninvasive assessment of the KRAS gene mutation status is helpful in guiding a targeted drug therapy and optimizing clinical decision-making (25). Cui et al. (12) extracted 960 radiological features from the T2-weighted imaging (T2WI) of 390 patients and identified 7 core features that significantly correlated with the cancer KRAS status after dimensionality reduction. The support vector machine (SVM) classification model constructed using these features achieved an area under the curve (AUC) of 0.714 in the external validation set. This study confirms the non-invasive preoperative evaluation of the KRAS gene expression in rectal cancer patients via radiological indicators, assisting in the development of individualized treatment strategies at the imaging level. Taguchi N (13) extracted 14 CT texture parameters from the portal vein phase CT images of primary tumours in rectal cancer patients. Moreover, the maximum standard uptake value (SUVmax) was obtained from 18F-FDG PET images and used as a quantitative parameter for metabolic activity. The univariate logistic regression method was applied to evaluate the predictive performance of each CT texture parameter and the SUVmax. On this basis, a comprehensive prediction model was constructed using multivariate SVM, and the AUC for the centralized prediction of KRAS mutations reached 0.82. However, owing to respiratory motion artefacts, the application of MRI in the abdomen has significant limitations. PET is expensive and involves ionizing radiation. The study of non-invasive prediction methods based solely on CT has an important clinical significance. In this study, we analysed the preoperative CT imaging data of numerous patients and used radiomics technology to extract a series of quantitative parameters, including morphology and texture features, to construct various prediction models. In terms of key indicators for predicting the KRAS mutation status, such as the area under the curve (AUC), accuracy, sensitivity and specificity, the XB model, which is based on the VP images, demonstrated an excellent performance and significantly outperformed the other models. The XB model has several advantages. It can prevent overfitting and enhance the generalization ability via regularization and pruning. It supports parallel and distributed computing and can efficiently process large-scale data. It can automatically adapt to sparse and missing data, enabling custom loss functionality. It is interpretable and has flexible parameter adjustments, which helps optimize the model performance. The traditional detection of the KRAS mutation status often requires invasive tissue biopsy, which has a certain risk of complications (26, 27). The noninvasive prediction ability of our methods is highly important in clinical applications, as it greatly improves the convenience and safety. Doctors can develop accurate personalized treatment plans for targeted therapy drugs based on the KRAS mutation status of patients to improve the treatment efficacy and patient prognosis.

In this study, we also investigated the predictive ability of radiomics models based on non-enhanced CT images for detecting KRAS mutations. Contrast agents can enhance the contrast between tissues, reveal the blood supply of tumours, and make lesion boundaries and internal structures clearer (28). Without contrast agents, the lesion display is poor, and lesions with a similar density to the surrounding tissues are difficult to identify, which may cause difficulties for models in learning lesion features and prevent them from accurately capturing key information related to KRAS mutations. Our results revealed that among the various models, the ET model based on NP-phase images had the best clinical effect, with an AUC value of 0.74. These results indicate that using plain scan CT images and radiomics is also highly important, as these methods can effectively help predict KRAS mutations in clinical practice. For patients who are unsuitable for contrast agents, plain scanning can be used as an alternative.

There is a close relationship between the traditional imaging descriptions (such as irregular shapes and the uneven enhancement of tumours) and radiomic features (29, 30). For example, the lobulation sign of a tumour corresponds to the shape features in radiomics. The uneven enhancement in enhanced scanning is closely related to the parameters of the grey level co-occurrence matrix in texture features. Compared with traditional imaging-based visual judgement, radiomics can capture subtle differences that are difficult for the naked eye to recognize via a quantitative analysis. In this study, eight radiomic features were retained for model construction after the feature screening process to ensure a close correlation between the feature parameters and the KRAS mutation status. The five first-order features describe the intensity distribution of images at the individual pixel level, which reflect the cell density and blood supply characteristics of tumours (31). The other three texture features of the GLCM, GLRM, and GLSZM mainly reflect the spatial relationships and texture information between pixels in the images (32). They provide a microscopic description of the internal structural heterogeneity of tumours (33). These radiomic features provide complete tumour information that cannot be obtained via quantitative visualization and are highly important for identifying the KRAS mutation status.

This study had several limitations. First, it was a retrospective study, and there may be biases in sample selection. Our model needs to be further validated via prospective studies in the future. Second, our data was from a single institution; moreover, it was obtained from two different CT scanners, and the scanning protocols were not identical. This may lead to differences in radiomics and affect the generalization ability of the model. Finally, manually delineating ROIs is time-consuming, and in the future, automated or semi-automated tool need to be developed to achieve an effective and automated tumour segmentation.

## Conclusion

In summary, this study demonstrated that radiomics methods based on CT images of the VP can accurately predict the KRAS gene mutation status in patients with colon cancer. This technique avoids the potential risks of tumour rupture and dissemination during biopsy and can serve as a powerful tool to help doctors develop personalized and precise targeted treatments for colorectal cancer, which is highly important in clinical work.

## Data Availability

The data analyzed in this study is subject to the following licenses/restrictions: The datasets used and/or analyzed during the current study are available from the corresponding author on reasonable request. Requests to access these datasets should be directed to Chuanming Li, licm@cqu.edu.cn.
