# Corrigendum: Prediction of KRAS gene mutations in colorectal cancer using a CT-based radiomic model

**DOI:** 10.3389/fmed.2025.1630007

**Published:** 2025-06-24

**Authors:** Wenjing Wang, Qingbiao Zhang, Shimei Fan, Yuyin Wang, Xingyan Le, Min Ai, Chunqi Du, Junbang Feng, Chuanming Li

**Affiliations:** ^1^Medical Imaging Department, Chongqing Emergency Medical Center, Chongqing University Central Hospital, School of Medicine, Chongqing University, Chongqing, China; ^2^Physical Examination Center, Chongqing Emergency Medical Center, Chongqing University Central Hospital, School of Medicine, Chongqing University, Chongqing, China; ^3^Department of Anesthesiology, Nanan District People's Hospital of Chongqing, Chongqing, China

**Keywords:** colorectal cancer, KRAS gene mutation, computed tomography, radiomics, machine learning

In the published article, there was an error in the author list. The author list was displayed as “Wenjing Wang^1^, Qingbiao Zhang^1^, Shimei Fan^2^, Yuyin Wang^1^, Xingyan Le^1^, Min Ai^3^, Chunqi Du^1*^, Junbang Feng^1*^ and Chuanming Li^1*^.” Wenjing Wang, Qingbiao Zhang and Shimei Fan made equally important contributions to this study. Therefore, the three authors share the first author status. The corrected author list appears below.

“Wenjing Wang^1†^, Qingbiao Zhang^1†^, Shimei Fan^2†^, Yuyin Wang^1^, Xingyan Le^1^, Min Ai^3^, Chunqi Du^1*^, Junbang Feng^1*^ and Chuanming Li^1*^.”

^†^These authors have contributed equally to this work.

The original article has been updated.

